# Is hypertension associated with arthritis? The United States national health and nutrition examination survey 1999–2018

**DOI:** 10.1080/07853890.2022.2089911

**Published:** 2022-07-04

**Authors:** Xiaopeng Liang, Oscar Hou In Chou, Ching Lung Cheung, Bernard M. Y. Cheung

**Affiliations:** a Division of Clinical Pharmacology and Therapeutics, Department of Medicine, School of Clinical Medicine, The University of Hong Kong, Hong Kong, China; b State Key Laboratory of Pharmaceutical Biotechnology, The University of Hong Kong, Pokfulam, Hong Kong, China; c Department of Pharmacology and Pharmacy, Li Ka Shing Faculty of Medicine, The University of Hong Kong, Hong Kong, China; d Institute of Cardiovascular Science and Medicine, The University of Hong Kong, Hong Kong, China

**Keywords:** Hypertension, arthritis, rheumatoid arthritis, osteoarthritis, NHANES

## Abstract

**Objective:**

Hypertension and arthritis are two common diseases in the general population, with multiple common risk factors. This study aimed to assess the association between hypertension (HTN) and arthritis.

**Patients and methods:**

This cohort study included 48,372 eligible non-pregnant participants aged ≥ 20 years who had valid data on hypertension and arthritis from the National Health and Nutrition Examination Survey (NHANES) 1999–2018. The association between hypertension and arthritis was studied by logistic regression, adjusting for demographics, socioeconomic factors, excess sodium intake, physical activity, ever smoking, diabetes status and body mass index (BMI).

**Results:**

Among the participants, 48.0% (95% CI: 47.2–48.9) had hypertension and 24.5% (95% CI: 23.8–25.3) had self-reported arthritis. Hypertension was associated with arthritis [OR = 2.90, (95% CI: 2.74–3.07), *p* < 0.01], which remained significant [OR = 1.27, (95% CI:1.18–1.37), *p* < 0.01] after adjustments. Stratified by the types of arthritis, the association remained significant in rheumatoid arthritis (RA) [OR = 1.25, (95% CI: 1.11–1.41), *p* < 0.01] and osteoarthritis (OA) [OR = 1.32, (95% CI: 1.16–1.50); *p* < 0.01]. There was no clear association between hypertension and OA in participants aged 60 years old and above [OR = 1.08, (95% CI: 0.92–1.26); *p* = 0.37].

**Conclusions:**

In this large nationally representative survey over 20 years, arthritis, including both RA and OA, was strongly associated with hypertension. Our study demonstrates a need for hypertension screening and blood control among patients with arthritis.

**Key Messages**
- Arthritis was associated with hypertension.
- Both rheumatoid arthritis and osteoarthritis are strongly associated with hypertension.
- There is an urgency for hypertension screening and blood control among patients with arthritis.

## Introduction

Hypertension increases the risk of morbidity and mortality globally. About 45.6% of adults in the United States [1] and 1.13 billion worldwide have hypertension [2]. It contributes to cardiovascular diseases, such as coronary heart disease and stroke [3,4]. Recent studies showed that hypertensive patients are more vulnerable to developing severe coronavirus disease 2019 (COVID-19) manifestations [5,6]. Control of hypertension helps to reduce cardiovascular mortality and morbidity [7].

Arthritis is another common chronic disease in the general population. Indeed, over half of the arthritis patients have hypertension [8]. As the proportion of US adults with arthritis has been predicted to rise to 49% by 2040, it is important to address the problems associated with arthritis [9]. There is accumulating evidence that arthritis, including rheumatoid arthritis (RA) and osteoarthritis (OA), is correlated with increased morbidity and mortality from cardiovascular events, with a relative risk from 1.49 to 4.31 [10,11]. Although an abundance of literature has examined the relationship between cardiovascular mortality and arthritis [10,12], few have investigated the link between hypertension and arthritis specifically. Hypertension and arthritis may share common pathophysiological pathways, such as chronic inflammation [13] and arterial stiffness [14]. Moreover, polypharmacy in arthritis, such as non-selective NSAID and cyclo-oxygenase II inhibitors usages, may compromise blood pressure control [15].

The prevalence of hypertension among arthritis patients varies from 3.8% [16] to 72.9% [17] in different reports. Several studies revealed the association between systolic blood pressure and OA; however, such association diminished after adjustment for overweight [18]. Some studies also found that hypertension was more common among RA patients [17]. Given the intermingling relationship between hypertension and other comorbidities, guidelines [19,20] recently called attention to optimising hypertension control to manage the comorbidities. Therefore, understanding the relationship between hypertension and arthritis would pave the way for more holistic management of both conditions.

This study aimed to use the data from US National Health and Nutrition Examination Survey (NHANES) 1999–2018 to investigate the association between hypertension and arthritis.

## Patients and methods

### Study population

**Figure 1.**
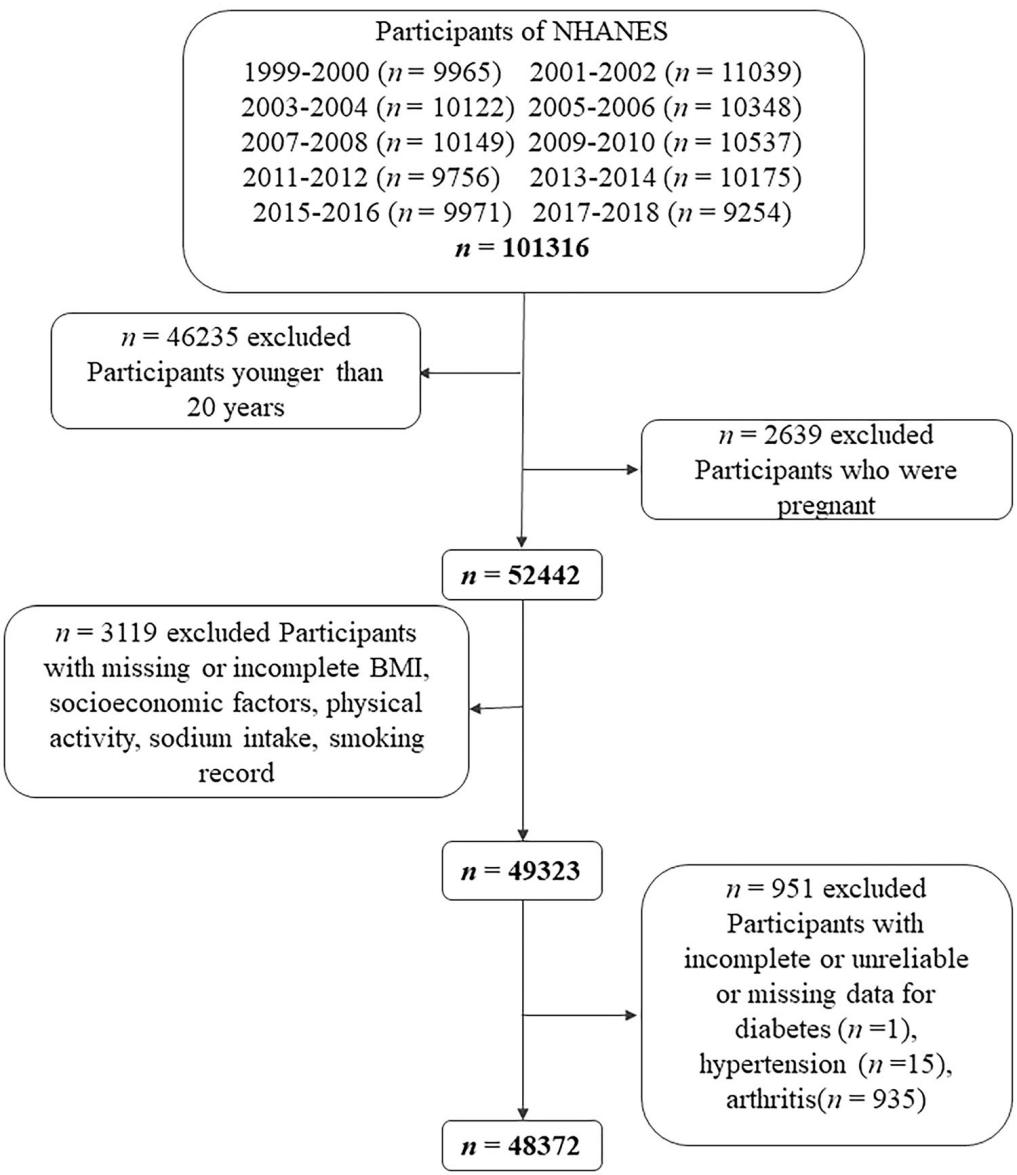
Flow chart of the screening process for the selection of eligible participants in NHANES 1999–2018.

NHANES is a continuous longitudinal survey dedicated to evaluating US health and nutritional status. The NHANES study was approved by the National Centre for Health Statistics Ethics Review Board in the US. Written consents were obtained from all adult participants before participation in the survey. A total of 101,316 individuals participated in NHANES 1999–2018. Participants who aged less than 20 years old (*n* = 46,235), pregnant women (*n* = 2,639), with missing or incomplete socioeconomic factors (education level, family income level, occupation, health insurance), body mass index, smoking records, dietary habits or physical activity were excluded (*n* = 3119). We also excluded incomplete, unreliable or uncertain data for diabetes (*n* = 1), hypertension (*n* = 15) and arthritis (*n* = 935). In the end, this study consisted of 48,372 eligible participants (Figure 1). Each participant represented approximately 50,000 individuals. The demographics, examination results, medical condition and dietary questionnaire, and laboratory data of the participants were extracted [21].

### Definition

According to the self-reported demographic questionnaires, race/ethnicity was classified into four groups, namely non-Hispanic whites, non-Hispanic blacks, Mexican Americans and others. The socioeconomic factors, including healthcare access, health insurance coverage, education level, or employment status, were defined. Education levels were categorized into high school or below, college and college graduates or above. According to the income-to-poverty ratio, family income was categorized as less than 130%, 131%–338% and more than 339%. The occupation was categorized into employment and unemployment. Health insurance was categorized into private health insurance, public health insurance only and no health insurance. The 2015–2020 Dietary Guidelines for Americans recommend consuming less than 2300 mg of dietary sodium per day for persons aged ≥14 years [22]. We defined over 2300 mg dietary sodium per day as excess sodium intake. Meanwhile, we defined those with no reported leisure time physical activity as physically inactive. Active groups were categorized as moderate activity or vigorous activity. Moderate activity was defined according to self-reported leisure time moderate activity (METs ranging from 3 to 6) five or more times per week. Vigorous activity was defined (MET > 6) three or more times per week. Insufficient activity was defined as those who were not inactive but did not meet the criteria for recommended levels of physical activity. The ever smoker status was defined as used to smoke over 100 cigarettes in their lifetime but was no longer smoking. Participants were stratified into the following groups: 20–39 years old, 40–59 years old and ≥ 60 years old. Participants with BMI over 25.0 were considered overweight, and over 30.0 as obese. Diabetes was defined either as a self-reported physician diagnosis of diabetes or elevated levels of fasting glucose [≥7.0 mmol/L (≥126 mg/dL)]), HbA1c (≥6.5%), or non-fasting glucose [≥11.1 mmol/L (200 mg/dL)]. According to the American Heart Association/American College of Cardiology (AHA/ACC) 2017 guideline [19], participants with systolic blood pressure ≥ 130 mmHg or diastolic blood pressure ≥ 80 mmHg were defined as hypertension. If the blood pressure measurement data were missing, patients with antihypertensive medication prescription in the clinical history "Are you now taking prescribed medicine." were also considered hypertensive. The Seventh Report of the Joint National Committee on Prevention, Detection, Evaluation and Treatment of High Blood Pressure (JNC 7) defined hypertension as systolic blood pressure ≥ 140 mmHg or diastolic blood pressure ≥90 mmHg, with self-reported physician diagnosis or with antihypertensive medications administrations. Arthritis was defined according to the question "Doctor ever said you had arthritis." correspondingly in the medical condition questionnaire [21]. Arthritis was further classified into RA, OA and other types of arthritis.

### Statistical analysis

Statistical analysis was conducted using STATA (version 15.1). Complex sample weights were applied to address the bias of sample inclusion selection, oversampling and nonresponse. The demographic characteristics and the overall prevalence of hypertension, diabetes, arthritis, overweight and obesity were estimated using a complex sample weight. The time trend of the prevalence of hypertension, arthritis, overweight and arthritis was assessed with adjustment of age, gender and race. Categorical variables were compared by Chi-square. Logistic regression models were used for covariates adjustments. Odds ratios (OR) with 95% CIs and *p* values were calculated. A two-tailed test with *p* values less than.05 was considered significant.

## Results

### Characteristics of study participants

Altogether, 48,372 eligible participants [mean age (±SE) 47.28 ± 0.18; 49.3% (95% CI: 48.9–49.7) male] were included in this analysis. The characteristics of the weighted percentages are summarized in Supplementary Table 1. This sample represented 204,148,270 noninstitutionalized adults in the US; 48.0% (95% CI: 47.2–48.9) of adults had hypertension, and the prevalence of arthritis was 24.5% (95% CI: 23.8–25.3). Over the 20-year period, there was a significant upward trend in the prevalence of OA from 5.7% (95% CI: 4.7–6.6) in 1999–2000 to 13.9% (95% CI: 12.0–16.0) in 2017–2018 (*p* < 0.01),

The characteristics of all 48,372 participants among individuals with and without hypertension are shown in Table 1. Participants with hypertension were older [mean age (±SE) 54.65 ± 0.22 vs. 40.47 ± 0.19, *p* < 0.01] compared with individuals without hypertension. A larger proportion of men [52.0% (95% CI: 51.2–52.8)] had hypertension compared with women [48.0% (95% CI: 47.2–48.8)]. There were significant race/ethnicity differences between participants with and without hypertension. The prevalence of ever smoking status, overweight/obesity, inactive physical activity and diabetes was higher among hypertensive participants than non-hypertensive participants (all *p* < 0.01). Compared with adults without hypertension, adults with hypertension consumed slightly less dietary sodium. The prevalence of arthritis among hypertensive subjects was more than twice that of their counterparts [35.1% (95% CI: 34.2–36.1) vs. 15.7% (95% CI: 14.9–16.4)]. Upon stratification, the prevalence of RA, OA and other arthritis was also higher among hypertensive participants than the non-hypertensive participants.

**Table 1.** Baseline characteristics of hypertension group versus the non-hypertensive group.

Characteristics	Hypertension	Non-hypertension	*p* value
N*	25,474	22,898	
Age†	54.65 ± 0.22	40.47 ± 0.19	<.01
Gender			
Men	52.0 (51.2–52.8)	46.8 (46.1–47.5)	<.01
Women	48.0 (47.2–48.8)	53.2 (52.5–53.9)	<.01
Race/Ethnicity			
Non-Hispanic White	69.9 (67.8–72)	66.8 (64.6–68.8)	<.01
Non-Hispanic Black	13.0 (11.7–14.4)	9.4 (8.5–10.3)	<.01
Mexican Americans	10.7 (9.5–12.2)	16.4 (14.8–18.2)	<.01
Others	6.3 (5.7–7.0)	7.4 (6.7–8.2)	<.01
Socioeconomic Factors			
Education level			
High school or below	45.1 (43.7–46.5)	38 (36.5–39.6)	<.01
Some College	30.3 (29.4–31.1)	31.1 (30.1–32.1)	.03
College graduate or above	24.7 (23.3–26.1)	30.9 (29.2–32.6)	<.01
Poverty to income ratio			
≤130%	19.1 (18–20.2)	20.4 (19.4–21.6)	.07
131%1–338%	39.8 (38.7–40.9)	37.8 (36.7–39)	.08
≥339%	41.2 (39.6–42.7)	41.7 (40.1–43.4)	.08
Health insurance status			
No	13.9 (13–14.8)	21.8 (20.8–23)	<.01
Public	23.6 (22.7–24.4)	14.6 (13.9–15.4)	<.01
Private	62.6 (61.3–63.8)	63.5 (62.2–64.9)	.07
Under Employment	55.3 (54.2–56.5)	72.3 (71.4–73.2)	<.01
BMI†	30.27 ± 0.08	27.35 ± 0.07	<.01
Overweight/Obesity	77.8 (77–78.6)	60.4 (59.3–61.6)	<.01
Physical activity			
Inactive	50.5 (49.4–51.5)	45.5 (44.4–46.7)	<.01
Moderate	33.1 (32–34.2)	34.9 (33.8–36)	.04
Vigorous	16.4 (15.6–17.2)	19.5 (18.6–20.5)	<.01
Excess Sodium Intake	83.4 (77.3–89.2)	88.5 (86.4–91.7)	.18
Ever smoking	29.9 (29–30.9)	20.0 (19.2–20.9)	<.01
Diabetes	17.5 (16.9–18.2)	4.8 (4.4–5.1)	<.01
Overall Arthritis	34.4 (33.5–35.4)	15.4 (14.6–16.1)	<.01
RA	8.8 (8.2–9.3)	3.7 (3.4–4.1)	<.01
OA	11.9 (11.2–12.6)	4.8 (4.4–5.2)	<.01
Other	13.8 (13.2–14.4)	6.9 (6.4–7.4)	<.01

BMI: body mass index; RA: Rheumatoid arthritis; OA: Osteoarthritis.

*N represents unweighted number, and the remaining values are weighted values using NHANES MEC examination weight.

†Figures are expressed as mean ± standard error (for mean age, BMI), other figures are expressed as percent (95% confidence intervals).

The characteristics of all 48,372 participants with self-reported RA, OA, other arthritis and no arthritis are illustrated in Table 2. Compared with the individuals without arthritis, participants with arthritis, which included RA, OA and other types of arthritis, were older (all *p* < 0.01]. A higher percentage of women [60.4% (95% CI: 59.4–61.4)] had arthritis compared with men [39.6% (95% CI: 38.6–40.6)]. This trend was also found in each type of arthritis. The proportion of non-Hispanic white participants was greater among participants with arthritis than among participants without arthritis [76.9% (95% CI: 74.9–78.7) vs. 65.4% (95% CI: 63.3–67.5)]. Participants with arthritis had a higher prevalence of ever smoking status, inactive physical activity, excess sodium intake, diabetes and overweight/obesity (all *p* < 0.01). The prevalence of hypertension was higher among arthritis participants than participants without arthritis [67.5% (95% CI: 66.2–68.7) vs. 41.6% (95% CI: 40.7–42.4)]. This trend was observed in RA, OA and other types of arthritis (all *p* < 0.01).

**Table 2.** Baseline characteristics of arthritis group versus the non-arthritis group.

	Arthritis				Non-Arthritis	*p* value
Characteristics	Total	RA	OA	Other		
N*	12884	3247	3962	5675	35488	
Age†	59.67 ± 0.19	66.77 ± 0.32	60.81 ± 0.27	58.08 ± 0.29	43.26 ± 0.17	<.01
Gender						
Men	39.6 (38.6–40.6)	36.1 (33.9–38.3)	36.8 (35–38.7)	43.9 (42.3–45.5)	52.5 (51.9–53)	<.01
Women	60.4 (59.4–61.4)	63.9 (61.7–66.1)	63.2 (61.3–65)	56.1 (54.5–57.7)	47.5 (47–48.1)	<.01
Race/Ethnicity						
Non-Hispanic White	76.9 (74.9–78.7)	79.8 (77.4–81.9)	77.7 (75.4–79.8)	74.3 (72–76.6)	65.4 (63.3–67.5)	<.01
Non-Hispanic Black	10.2 (9.1–11.5)	8.6 (7.3–10.1)	9.7 (8.4–11.1)	11.6 (10.3–13.1)	11.4 (10.3–12.6)	<.01
Mexican Americans	8.0 (6.8–9.2)	6.9 (6–8.1)	7 (5.9–8.2)	9.5 (7.9–11.3)	15.6 (14.1–17.3)	<.01
Others	5.0 (4.4–5.6)	4.7 (3.7–6)	5.7 (4.7–6.8)	4.6 (3.9–5.4)	7.5 (6.9–8.3)	<.01
Socioeconomic Factors						
Education level						
High school or below	46.5 (44.8–48.3)	44.7 (42–47.5)	40.7 (38.3–43.1)	52.4 (50.1–54.6)	39.7 (38.4–41.1)	<.01
Some College	30.7 (29.5–32)	31.7 (29.5–34.1)	32.7 (30.7–34.7)	28.5 (26.9–30.3)	30.7 (29.9–31.5)	.16
College graduate or above	22.7 (21.1–24.5)	23.5 (21.3–25.9)	26.7 (24.2–29.3)	19.1 (17–21.4)	29.6 (28.1–31.1)	<.01
Poverty to income ratio						
≤130%	20.9 (19.5–22.4)	19 (16.8–21.5)	20.5 (18.7–22.5)	22.3 (20.7–24)	19.4 (18.4–20.4)	.07
131%-338%	40.3 (39–41.6)	40.3 (37.8–42.9)	38.4 (36.2–40.7)	41.8 (39.9–43.7)	38.3 (37.3–39.2)	.04
≥339%	38.8 (37–40.7)	40.7 (37.8–43.6)	41.1 (38–44.2)	35.9 (33.6–38.3)	42.3 (40.8–43.8)	<.01
Health insurance status						
No	8.9 (8.1–9.8)	7.3 (6.2–8.7)	8.3 (7.3–9.6)	10.3 (9.2–11.6)	21 (20–22)	<.01
Public	30.5 (29.3–31.8)	30.6 (28.4–32.9)	31.8 (29.8–33.9)	29.5 (27.8–31.2)	15.1 (14.5–15.8)	<.01
Private	60.6 (59–62)	62.1 (59.5–64.6)	59.8 (57.5–62.1)	60.2 (58.3–62.1)	63.9 (62.7–65.1)	<.01
Under Employment	42.2 (40.8–43.7)	42.2 (39.6–44.8)	38.9 (36.5–41.3)	45 (43.1–46.8)	71.3 (70.5–72.1)	<.01
BMI†	30.54 ± 0.09	30.16 ± 0.18	30.81 ± 0.19	30.55 ± 0.12	28.17 ± 0.07	<.01
Overweight/Obesity	77.7 (76.8–78.6)	76.5 (74.4–78.5)	78 (76.2–79.7)	78.1 (76.7–79.5)	65.8 (64.9–66.7)	<.01
Physical activity						
Inactive	54 (52.6–55.4)	52.7 (50–55.4)	56 (53.8–58.1)	53.1 (51.2–55.1)	45.9 (44.9–47)	<.01
Moderate	31.8 (30.5–33)	34.3 (31.6–37)	29.4 (27.7–31.2)	32.1 (30.4–33.9)	34.8 (33.8–35.8)	<.01
Vigorous	14.2 (13.2–15.3)	13 (11.3–14.8)	14.6 (12.9–16.4)	14.7 (13.2–16.4)	19.3 (18.5–20.1)	<.01
Excess Sodium Intake	88.2 (86.1–89.4)	90.2 (87.1–93.6)	87.2 (86.1–89.3)	86.1 (84.2–88.2)	80.5 (79.7–81.8)	<.01
Ever smoking	33.8 (32.6–34.9)	35.4 (33.3–37.5)	34.1 (31.7–36.5)	32.3 (30.6–34.1)	21.8 (21.1–22.5)	<.01
Diabetes	19.6 (18.6–20.6)	17.8 (16.3–19.3)	20.9 (19.1–22.9)	19.8 (18.3–21.4)	8.1 (7.7–8.4)	<.01
Hypertension	67.5 (66.2–68.7)	68.7 (66.2–71)	69.7 (67.6–71.7)	65 (63–66.9)	41.6 (40.7–42.4)	<.01

BMI: body mass index; RA: Rheumatoid arthritis; OA: Osteoarthritis.

*N represents unweighted number, and the remaining values are weighted values using NHANES MEC examination weight.

†Figures are expressed as mean ± standard error (for mean age, BMI), other figures are expressed as percent (95% confidence intervals).

### The association between hypertension and arthritis

Table 3 shows the logistic regression analysis of arthritis before and after adjustments for the covariates. Overall, arthritis is significantly associated with hypertension [OR = 2.90 (95% CI: 2.74–3.07)]. After adjusting for demographics, socioeconomic factors, ever smoking, excess sodium intake, physical activity, diabetes status and BMI, the association remained significant [OR = 1.27 (95% CI: 1.18–1.37), *p* < 0.01]. Both RA [OR = 2.50 (95% CI: 2.25–2.78)] and OA [OR = 2.68 (95% CI: 2.42–2.98)] were significantly associated with hypertension. The competing risk analysis of the association between hypertension and RA/OA is shown in Supplementary Table 2. After adjustments, both RA [OR = 1.25 (95% CI: 1.11–1.41)] and OA [OR = 1.32 (95% CI: 1.16–1.50)] remained significantly associated with hypertension (Table 4). The association was significant both for men and women among OA participants, whereas there was no significant association in RA among men [OR = 1.07 (95% CI: 0.88–1.31), *p* = 0.48]. In the 20–39 [OR = 1.47 (95% CI: 1.22–1.77)] and 40–59 [OR: 1.34 (95% CI: 1.22–1.48)] age group, arthritis was significantly associated with hypertension (Supplementary Table 3). In RA, the association was significantly amongst participants who were 20–39 years old [OR =1.63 (95% CI: 1.13–2.36)] and equalled or over 60 years old [OR = 1.32 (95% CI: 1.13–1.54)]. Meanwhile, in OA and other arthritis, the association was significant among participants aged less than 60 years (Table 4).

**Table 3.** Association between hypertension and total arthritis.

	Crude OR	Model 1	Model 2	Model 3	Model 4
Total	2.9 (2.74–3.07)	1.44 (1.35–1.54)	1.39 (1.29–1.5)	1.29 (1.2–1.39)	1.27 (1.18–1.37)

Figures are expressed as odds ratio (95% confidence interval).

Model 1: Adjusted for age, gender and race.

Model 2: Further adjusted for socioeconomic factors including health insurance coverage, education level, employment status and poverty to income ratio.

Model 3: Further adjusted for physical activity, excess sodium intake, ever cigarette smoking and obesity.

Model 4: Further adjusted for diabetes.

**Table 4.** Subgroup analysis of the association of arthritis subtype (RA, OA, and others) and hypertension.

	RA		OA		Other	
	OR (95% CI)	*p* value	OR (95% CI)	*p* value	OR (95% CI)	*p* value
Overall	1.25 (1.11–1.41)	<.01	1.32 (1.16–1.5)	<.01	1.09 (0.99–1.21)	.07
Gender						
Men	1.07 (0.88–1.31)	.48	1.25 (1.02–1.52)	.03	1.16 (0.99–1.36)	.06
Women	1.39 (1.19–1.62)	<.01	1.37 (1.16–1.62)	<.01	1.06 (0.93–1.2)	.41
Age						
20–39 y	1.63 (1.13–2.36)	<.01	1.72 (1.26–2.35)	<.01	1.37 (1.07–1.76)	.01
40–59 y	1.16 (0.96–1.39)	.12	1.56 (1.3–1.87)	<.01	1.24 (1.05–1.46)	.01
≥ 60 y	1.32 (1.13–1.54)	<.01	1.08 (0.92–1.26)	.37	0.9 (0.78–1.04)	.15

RA: rheumatoid arthritis; OA: osteoarthritis.

All data were adjusted for gender (except gender-specific estimates), age (except age-specific estimates), race, health insurance coverage, education level, employment status and poverty to income ratio, physical activity, excess sodium intake, ever cigarette smoking, obesity and diabetes.

## Discussion

Our study showed a robust association between hypertension and arthritis using 20 years of nationally representative data from NHANES. Furthermore, this association remained significant after adjusting the common factors between hypertension and arthritis, including gender, race, age, socioeconomic factors, excess sodium intake, physical activity, overweight or obese status, ever smoking status and diabetes. Given the large sample and the reasonable quality control, our analysis should be reliable.

Several studies [8,17] revealed that the prevalence of hypertension in arthritis is higher than in the general population. In this study, hypertension was reported in 67.5% (95% CI, 66.2–68.7) arthritis patients; this was much higher than the 41.6% in the non-arthritic participants and 48.0% in the general population. It was proposed that inflammation [13,16], physical inactivity [23] and medications [15] may contribute to this interrelationship. Firstly, inflammatory mediators, such as chemokines and cytokines [24], have been implicated as a predictor of endothelial dysfunction and arterial stiffness [16], which play a pivotal part in the pathogenesis of arthritis and hypertension. Secondly, arthritis patients may have reduced fitness and activities owing to pain, joint damage and the fear of deterioration. Nevertheless, the American College of Rheumatology guideline [23] suggests that physical exercise effectively maintains the well-being of arthritis patients. Consequently, this physical inactivity contributes to the development of physiological impairment, including endothelial dysfunction, one of the common pathophysiological changes for hypertension and arthritis [25]. Moreover, polypharmacy in arthritis [15], including the frequent usage of non-selective NSAIDs for analgesics, may cause hypertension and compromise blood pressure control.

In the age-stratified analysis, there was a strong association between hypertension and arthritis (Table 4, Supplementary Table 3). However, the association between hypertension and arthritis did not increase with age; instead, it diminished among older participants. This indicates that the association is not merely a consequence of ageing. Indeed, despite the increasing prevalence of arthritis among the elderly, it was predicted that adults aged 18–65 will account for the majority of arthritis cases by 2040 (67%) [9]. The lower blood pressure control rate among young adults could partially explain the strong association between hypertension and arthritis [26,27]. As the prevalence of hypertension was the lowest in young adults [28], the blood pressure control rate in young adults (38%) was also much lower than those in their middle (55%) and older age (53%) [27,29]. Young adults are also over 40% slower than middle-aged and older adults in terms of medication initiation [26]. Another plausible explanation could be that different forms of arthritis have different ages of onset: osteoarthritis typically occurs after middle age; the prevalence of rheumatoid arthritis in each age group is similar, while the prevalence of fibromyalgia increases with age. In our study, among the 12,884 arthritis patients, 3962 [33.5% (95% CI, 32.0–35.1)] had OA, 3247 [25.0% (95% CI, 23.7–26.3)] had RA, 5675 [41.5% (95% CI, 40.2–42.9)] had arthritis not classified. Consistently, the association between hypertension and OA remained significant among participants aged less than 60 years.

In the sex-stratified analysis, we found that the association in women between hypertension and arthritis, especially both RA and OA, was significant after adjustment; in men, hypertension was not significantly associated with RA. Gender-related differences in hypertension are well described. Young men are more likely to have hypertension (27% in men vs. 12% in women) and lower hypertension awareness (25% in men and 32% in women) than women of the same age [28,30]. Meanwhile, there is no discrepancy between older men and women aged over 60 years regarding the prevalence of hypertension [31]. A Korean study revealed that among adults aged 60 years and above, men even have a lower prevalence of hypertension and better hypertension control than women [32]. Furthermore, women with declined oestrogen levels before menopause have an increased risk of high blood pressure [33]. In the United States, 75% of postmenopausal women have hypertension [34]. Lastly, arthritis is much more frequent among women, with a women-to-men ratio of 3:1 in RA [35] and 1.3:1 in OA [36]. These could potentially explain the diminished association in men aged over the 60-year group.

This study has some limitations that should be acknowledged. Firstly, the US NHANES is a cross-sectional survey; it does not provide longitudinal follow-up data. Future investigations are needed to demonstrate the causal relationship between arthritis and hypertension due to its retrospective nature. Besides, NHANES uses a self-reported questionnaire for some medication health variables, which would introduce recall and self-reported bias. Thirdly, medication usage was not considered in the study. For instance, NSAIDs usage was unavailable in NHANES from 1999 to 2018. As such, how different medication interactions affect the outcomes of hypertension and arthritis should be studied in future research.

## Conclusions

In conclusion, our analysis from the nationally-representative survey showed a strong association between hypertension and arthritis. In particular, RA and OA were significantly associated with hypertension. This suggested that clinicians should screen for hypertension among people with arthritis of all ages and intervene early.

## Supplementary Material

Supplemental Material

## Data Availability

The data that support the findings of this study are available in NHANES 1999-2018. These data were derived from the following sources available in public domain https://www.cdc.gov/nchs/nhanes/index.htm

## References

[1] Rana J, Oldroyd J, Islam MM, et al. Prevalence of hypertension and controlled hypertension among United States adults: evidence from NHANES 2017-18 survey. Int J Cardiol Hypertens. 2020;7:100061. 33447782 10.1016/j.ijchy.2020.100061 PMC7803033

[2] Mills KT, Bundy JD, Kelly TN, et al. Global disparities of hypertension prevalence and control: a systematic analysis of population-based studies from 90 countries. Circulation. 2016;134(6):441–450. 27502908 10.1161/CIRCULATIONAHA.115.018912 PMC4979614

[3] Joffres M, Falaschetti E, Gillespie C, et al. Hypertension prevalence, awareness, treatment and control in national surveys from England, the USA and Canada, and correlation with stroke and ischaemic heart disease mortality: a cross-sectional study. BMJ Open. 2013;3(8):e003423. 10.1136/bmjopen-2013-003423 PMC3758966 23996822

[4] Bidani AK, Griffin KA. Pathophysiology of hypertensive renal damage: implications for therapy. Hypertension. 2004;44(5):595–601. 15452024 10.1161/01.HYP.0000145180.38707.84

[5] Li HL, Cheung BMY. The proportion of adult Americans at risk of severe COVID-19 illness. J Gen Intern Med. 2021;36(1):259–261. 33104999 10.1007/s11606-020-06325-9 PMC7586862

[6] Savoia C, Volpe M, Kreutz R. Hypertension, a moving target in COVID-19: current views and perspectives. Circ Res. 2021;128(7):1062–1079. 33793331 10.1161/CIRCRESAHA.121.318054 PMC8011346

[7] Kahan T. Decisions about antihypertensive treatment should focus on reducing cardiovascular risk. Lancet. 2021;397(10285):1598–1599. 33933193 10.1016/S0140-6736(21)00877-1

[8] Weber MA. Treatment of patients with hypertension and arthritis pain: new concepts. Am J Med. 2009;122(5 Suppl):S16–S22. 19393822 10.1016/j.amjmed.2009.03.004

[9] Hootman JM, Helmick CG, Barbour KE, et al. Updated projected prevalence of Self-reported doctor-diagnosed arthritis and arthritis-attributable activity limitation among US adults, 2015-2040. Arthritis Rheumatol. 2016;68(7):1582–1587. 27015600 10.1002/art.39692 PMC6059375

[10] Wolfe F, Freundlich B, Straus WL. Increase in cardiovascular and cerebrovascular disease prevalence in rheumatoid arthritis. J Rheumatol. 2003;30(1):36–40. 12508387

[11] Assous N, Touzé E, Meune C, et al. Cardiovascular disease in rheumatoid arthritis: single-center hospital-based cohort study in France. Joint Bone Spine. 2007;74(1):66–72. 17174586 10.1016/j.jbspin.2006.10.001

[12] Ong KL, Wu BJ, Cheung BM, et al. Arthritis: its prevalence, risk factors, and association with cardiovascular diseases in the United States, 1999 to 2008. Ann Epidemiol. 2013;23(2):80–86. 23218811 10.1016/j.annepidem.2012.11.008

[13] Niskanen L, Laaksonen DE, Nyyssönen K, et al. Inflammation, abdominal obesity, and smoking as predictors of hypertension. Hypertension. 2004;44(6):859–865. 15492131 10.1161/01.HYP.0000146691.51307.84

[14] Klocke R, Cockcroft JR, Taylor GJ, et al. Arterial stiffness and Central blood pressure, as determined by pulse wave analysis, in rheumatoid arthritis. Ann Rheum Dis. 2003;62(5):414–418. 12695151 10.1136/ard.62.5.414 PMC1754549

[15] Morrison A, Ramey DR, van Adelsberg J, et al. Systematic review of trials of the effect of continued use of oral non-selective NSAIDs on blood pressure and hypertension. Curr Med Res Opin. 2007;23(10):2395–2404. 17714606 10.1185/030079907X219553

[16] Roman MJ, Devereux RB, Schwartz JE, et al. Arterial stiffness in chronic inflammatory diseases. Hypertension. 2005;46(1):194–199. 15911740 10.1161/01.HYP.0000168055.89955.db

[17] Chung CP, Oeser A, Solus JF, et al. Prevalence of the metabolic syndrome is increased in rheumatoid arthritis and is associated with coronary atherosclerosis. Atherosclerosis. 2008;196(2):756–763. 17266963 10.1016/j.atherosclerosis.2007.01.004

[18] Niu J, Clancy M, Aliabadi P, et al. Metabolic syndrome, its components, and knee osteoarthritis: the Framingham osteoarthritis study. Arthritis Rheumatol. 2017;69(6):1194–1203. 28257604 10.1002/art.40087 PMC5449217

[19] Whelton PK, Carey RM, Aronow WS, et al. 2017 ACC/AHA/AAPA/ABC/ACPM/AGS/APhA/ASH/ASPC/NMA/PCNA guideline for the prevention, detection, evaluation, and management of high blood pressure in adults: Executive summary: a report of the American College of Cardiology/American Heart Association Task Force on Clinical Practice Guidelines. Hypertension. 2018;71(6):1269–1324. 29133354 10.1161/HYP.0000000000000066

[20] Unger T, Borghi C, Charchar F, et al. 2020 International society of hypertension global hypertension practice guidelines. Hypertension. 2020;75(6):1334–1357. 32370572 10.1161/HYPERTENSIONAHA.120.15026

[21] Centers for Disease Control and Prevention (CDC). National center for health statistics (NCHS). national health and nutrition examination survey questionnaire, examination protocol and laboratory protocol. Hyattsville (MD): U.S. Department of Health and Human Services, Centers for Disease Control and Prevention; 1999–2018. Available from: https://www.cdc.gov/nchs/nhanes/index.htm.

[22] Jackson SL, King SM, Zhao L, et al. Prevalence of excess sodium intake in the United States - NHANES, 2009-2012. MMWR Morb Mortal Wkly Rep. 2016;64(52):1393–1397. 26741238 10.15585/mmwr.mm6452a1

[23] Guidelines for the management of rheumatoid arthritis: 2002 update. Arthritis Rheum. 2002;46(2):328–346. 11840435 10.1002/art.10148

[24] De Miguel C, Rudemiller NP, Abais JM, et al. Inflammation and hypertension: new understandings and potential therapeutic targets. Curr Hypertens Rep. 2015;17(1):507. 25432899 10.1007/s11906-014-0507-z PMC4418473

[25] Booth FW, Roberts CK, Laye MJ. Lack of exercise is a major cause of chronic diseases. Compr Physiol. 2012;2(2):1143–1211. 23798298 10.1002/cphy.c110025 PMC4241367

[26] Johnson HM, Thorpe CT, Bartels CM, et al. Antihypertensive medication initiation among young adults with regular primary care use. J Gen Intern Med. 2014;29(5):723–731. 24493322 10.1007/s11606-014-2790-4 PMC4000352

[27] Vital signs: prevalence, treatment, and control of hypertension–United States, 1999-2002 and 2005-2008. MMWR Morb Mortal Wkly Rep. 2011;60(4):103–108. 21293325

[28] Ong KL, Cheung BM, Man YB, et al. Prevalence, awareness, treatment, and control of hypertension among United States adults 1999-2004. Hypertension. 2007;49(1):69–75. 17159087 10.1161/01.HYP.0000252676.46043.18

[29] Go AS, Mozaffarian D, Roger VL, American Heart Association Statistics Committee and Stroke Statistics Subcommittee, et al. Heart disease and stroke statistics–2013 update: a report from the American Heart Association. Circulation. 2013;127(1):e6–e245. 23239837 10.1161/CIR.0b013e31828124ad PMC5408511

[30] Everett B, Zajacova A. Gender differences in hypertension and hypertension awareness among young adults. Biodemography Soc Biol. 2015;61(1):1–17. 25879259 10.1080/19485565.2014.929488 PMC4896734

[31] Brindel P, Hanon O, Dartigues JF, 3C Study Investigators, et al. Prevalence, awareness, treatment, and control of hypertension in the elderly: the three city study. J Hypertens. 2006;24(1):51–58. 16331101 10.1097/01.hjh.0000198028.84353.86

[32] Choi HM, Kim HC, Kang DR. Sex differences in hypertension prevalence and control: Analysis of the 2010-2014 Korea national health and nutrition examination survey. PLoS One. 2017;12(5):e0178334. 28542557 10.1371/journal.pone.0178334 PMC5444798

[33] Maas AH, Franke HR. Women's health in menopause with a focus on hypertension. Neth Heart J. 2009;17(2):68–72. 19247469 10.1007/BF03086220 PMC2644382

[34] Barton M, Meyer MR. Postmenopausal hypertension: mechanisms and therapy. Hypertension. 2009;54(1):11–18. 19470884 10.1161/HYPERTENSIONAHA.108.120022

[35] Quintero OL, Amador-Patarroyo MJ, Montoya-Ortiz G, et al. Autoimmune disease and gender: plausible mechanisms for the female predominance of autoimmunity. J Autoimmun. 2012;38(2-3):J109–19. 22079680 10.1016/j.jaut.2011.10.003

[36] Zhang Y, Jordan JM. Epidemiology of osteoarthritis. Clin Geriatr Med. 2010;26(3):355–369. 20699159 10.1016/j.cger.2010.03.001 PMC2920533

