# Supplementary material for: Is hypertension associated with arthritis? The United States national health and nutrition examination survey 1999–2018
Source: Ann Med. 2022 Jul 4;54(1):1767–75. doi: 10.1080/07853890.2022.2089911 (PMC9258429; doi:10.1080/07853890.2022.2089911)
Supplement: Supplemental Material [file IANN_A_2089911_SM4387.docx]

**Supplementary Table 1.** **Characteristics of participants included in this study**

|  | 1999-2000 | 2001-2002 | 2003-2004 | 2005-2006 | 2007-2008 | 2009-2010 | 2011-2012 | 2013-2014 | 2015-2016 | 2017-2018 | P for linear trend | Overall |
| --- | --- | --- | --- | --- | --- | --- | --- | --- | --- | --- | --- | --- |
| N* | 4013 | 4334 | 3489 | 4291 | 5510 | 5867 | 5123 | 5397 | 5275 | 5073 |  | 48732 |
| Age† | 46.46 ± 0.39 | 46.04 ± 0.48 | 46.43 ± 0.59 | 47.00 ± 0.73 | 46.90 ± 0.44 | 47.23 ± 0.50 | 47.57 ± 0.84 | 47.73 ± 0.37 | 48.27 ± 0.59 | 48.45 ± 0.57 | .02 | 47.28 ± 0.18 |
| Men (%) | 50.0 (48.4-51.7) | 49.4 (48.2-50.6) | 50.8 (48.8-52.8) | 49.7 (48.6-50.9) | 48.9 (47.7-50) | 49.0 (48.0-50.1) | 49.0 (47.3-50.6) | 49.0 (47.8-50.2) | 48.9 (47.7-50.1) | 48.8 (47.1-50.6) | .82 | 49.3 (48.9-49.7) |
| Race/Ethnicity (%) | | | | | | | | | | | | |
| Non-Hispanic White | 70.0 (64.4-75.1) | 73.4 (68.5-77.7) | 72.1 (65-78.3) | 72.4 (66.7-77.5) | 69.8 (62.2-76.4) | 68.2 (61.3-74.4) | 66.8 (58.8-73.9) | 66.2 (59.5-72.3) | 64.1 (56.2-71.4) | 62.8 (57.6-67.7) | .02 | 68.3 (66.2-70.3) |
| Non-Hispanic Black | 10.5 (7.7-14.1) | 10.3 (7.5-14.1) | 11.3 (8.0-15.6) | 11.3 (8.1-15.7) | 11.2 (8.0-15.4) | 11.3 (9.7-13.1) | 11.3 (7.6-16.5) | 11.2 (8.5-14.8) | 11.3 (7.7-16.2) | 11.3 (8.5-14.8) |  | 11.1 (10.0-12.3) |
| Mexican Americans | 15.3 (10.1-22.4) | 12.2 (8.3-17.5) | 11.6 (7.8-16.9) | 11.1 (8.7-14.2) | 13.0 (9.6-17.4) | 13.5 (8.8-20.1) | 14.2 (10-19.8) | 14.6 (10.5-20) | 15.1 (10.7-21.1) | 15.5 (12.1-19.6) |  | 13.7 (12.3-15.7) |
| Others | 4.2 (2.8-6.3) | 4.1 (2.9-5.7) | 5.0 (3.7-6.8) | 5.1 (4.2-6.2) | 6.0 (4.2-8.4) | 7.0 (5.1-9.5) | 7.7 (5.8-10.2) | 8.0 (6.7-9.5) | 9.5 (7.2-12.4) | 10.5 (8.2-13.2) |  | 6.9 (6.3-7.6) |
| Education level | | | | | | | | | | | | |
| High school or below | 51.2(47-55.4) | 44.1(40.9-47.5) | 46(42.9-49.1) | 42.6(38.9-46.4) | 45.8(41.1-50.5) | 41.7(39-44.5) | 36.8(31.9-42.1) | 37(32.9-41.2) | 35.2(30.7-40) | 38.1(34.1-42.2) |  | 41.4(40.1-42.8) |
| Some College | 26.9(24.9-28.9) | 29.5(27.2-31.8) | 31.2(28.9-33.6) | 31.4(29.4-33.4) | 28.9(27.2-30.7) | 30.3(28.7-32) | 32(29.1-35) | 32.7(30.6-34.9) | 32.4(29.7-35.3) | 30.8(28.3-33.4) |  | 30.7(30-31.4) |
| College graduate or above | 21.9(17.3-27.4) | 26.4(23.5-29.5) | 22.8(19.8-26.2) | 26(22.2-30.3) | 25.3(21.5-29.5) | 28(25.5-30.6) | 31.2(26.4-36.4) | 30.3(26.6-34.3) | 32.3(26.7-38.6) | 31.1(26.3-36.4) |  | 27.9(26.5-29.4) |
| Poverty to income ratio | | | | | | | | | | | | |
| ≤130% | 21(16.4-26.4) | 18.5(16.2-21) | 19.2(15.5-23.5) | 16.5(14.6-18.6) | 18.9(16.1-22.1) | 19.9(18-22) | 23.5(20.3-27.1) | 23(19-27.6) | 19.2(16.3-22.5) | 17.8(16.6-19.1) | <.01 | 19.8(18.8-20.8) |
| 131%-338% | 42.4(38.3-46.6) | 36.5(34.6-38.5) | 40.6(37.9-43.2) | 37.8(34.5-41.1) | 38.8(36.6-41) | 38.3(36.6-40.2) | 36.1(32.6-39.7) | 37.3(35.2-39.5) | 40.4(37.9-43) | 40.3(37.1-43.5) |  | 38.8(37.9-39.7) |
| ≥339% | 36.6(31.5-42) | 45(41.5-48.5) | 40.2(36.1-44.5) | 45.8(41.4-50.1) | 42.3(37.9-46.9) | 41.8(39.5-44) | 40.4(35-46) | 39.7(34.6-45.1) | 40.4(35.6-45.4) | 42(38.4-45.7) |  | 41.5(40-42.9) |
| Health insurance status | | | | | | | | | | | | |
| No | 19.6(17.1-22.3) | 18(15.9-20.2) | 19(17.4-20.6) | 19.1(15.6-23.2) | 19.5(17.7-21.6) | 20.8(18.8-23) | 20(17.4-22.9) | 18.1(15.8-20.5) | 13.5(11.1-16.4) | 13.8(10.5-18.1) | <.01 | 18(17.2-18.9) |
| Public | 13.2(11.3-15.3) | 13.9(12.8-15.1) | 18.2(15.9-20.7) | 16.4(14.6-18.2) | 15.4(13.3-17.7) | 16.4(14.7-18.3) | 19.2(16.5-22.2) | 20.4(18.2-22.8) | 25.1(22.1-28.3) | 27.8(25.6-30.1) |  | 18.9(18.2-19.6) |
| Private | 67.3(62.9-71.3) | 68.1(65.6-70.6) | 62.9(59.8-65.8) | 64.5(60.4-68.5) | 65.1(61.4-68.6) | 62.7(61.1-64.3) | 60.7(56.5-64.8) | 61.5(57.6-65.4) | 61.4(56.5-66.1) | 58.4(54.8-61.9) |  | 63.1(61.9-64.2) |
| Under Employment | 64.8(62.2-67.3) | 66.3(63.7-68.9) | 63.8(61.4-66.1) | 67.8(64.6-70.9) | 64.8(61.7-67.8) | 62.6(60.8-64.3) | 61.2(58.2-64.2) | 62.2(59.3-65.1) | 64.4(61-67.7) | 64.3(62.1-66.4) | 0.67 | 64.2(63.3-65) |
| Physical activity | | | | | | | | | | | | |
| Inactive | 41.9(38.4-45.5) | 34.1(31.6-36.8) | 34.1(31.7-36.6) | 32.7(29.9-35.7) | 52.8(50.3-55.4) | 58(55.1-60.8) | 59.9(57.3-62.5) | 59.6(57.1-62.2) | 50.9(47.7-54.1) | 47.1(44.4-49.7) | <.01 | 47.9(47-48.8) |
| Moderate | 42.6(38.3-46.9) | 52.1(48.8-55.3) | 57.9(55.7-60.1) | 59.7(56.6-62.8) | 25.8(23.7-28) | 22.2(20.8-23.6) | 21.1(19.2-23) | 21.1(19.3-23.1) | 25.3(23.6-27.2) | 25.9(23.5-28.5) |  | 34.1(33.1-35) |
| Vigorous | 15.5(14-17.2) | 13.8(11.3-16.7) | 7.9(6.8-9.3) | 7.5(6.5-8.7) | 21.4(19.6-23.3) | 19.8(17.6-22.3) | 19(17-21.2) | 19.2(18.1-20.5) | 23.8(21.4-26.3) | 27(24.3-30) |  | 18(17.3-18.8) |
| Excess Na Intake | 88.3(87.2-89.7) | 88.2(87.1-89.4) | 87.6(86.3-88.9) | 87.2(86.1-87.5) | 87.5(86.3-88.7) | 87.4(86.2-88.6) | 86.4(85.3-87.7) | 87.6(86.4-88.8) | 87.6(86.5-88.9) | 87.3(86.2-88.7) | .17 | 87.8(87.4-98.2) |
| Overweight/obesity | 64.3 (61-67.4) | 65.2 (63.9-66.5) | 66.5 (64.5-68.4) | 67.1 (64.5-69.6) | 68 (66.3-69.7) | 69.1 (66.5-71.6) | 69 (65.8-72.1) | 70.7 (69.1-72.2) | 71.7 (68.5-74.6) | 73.7 (70.9-76.2) | <.01 | 68.8 (68.0-69.6) |
| Ever cigarette smoking (%) | 25.3 (23.2-27.6) | 25.4 (22.8-28.1) | 25.0 (23.3-26.9) | 25.0 (23-27.1) | 24.3 (22.9-25.6) | 24.5 (22-27.1) | 24.4 (21.9-27.1) | 23.7 (21.8-25.8) | 25.4 (23.3-27.7) | 25.1 (23.3-26.9) | .98 | 24.8 (24.1-25.5) |
| Systolic blood pressure† | 129.63 ± 1.24 | 127.32 ± 1.04 | 132.11 ± 1.65 | 128.04 ± 1.71 | 122.72 ± 0.40 | 122.83 ± 0.54 | 124.49 ± 0.74 | 125.41 ± 0.70 | 125.77 ± 0.62 | 128.33 ± 0.85 | <.01 | 126.45 ± 0.32 |
| Diastolic blood pressure† | 75.93 ± 0.60 | 74.84 ± 0.55 | 76.08 ± 0.78 | 73.32 ± 1.05 | 71.49 ± 0.41 | 70.60 ± 0.78 | 72.94 ± 0.65 | 71.68 ± 0.72 | 71.43 ± 0.57 | 75.33 ± 0.83 | <.01 | 73.23 ± 0.23 |
| Hypertension (2017ACC/AHA) | 51.9 (48.7-55.1) | 48.6 (45.7-51.4) | 49.9 (46.8-53) | 47.6 (45.5-49.8) | 44.5 (42.2-46.8) | 45.5 (42.7-48.3) | 48.2 (45.2-51.2) | 46.2 (44.4-48) | 48.4 (46.3-50.6) | 50.5 (47.8-53.2) | .05 | 48.0 (47.2-48.9) |
| Hypertension (JNC7) | 38.9 (35.8-42.1) | 36.8 (34.2-39.5) | 42.2 (39.2-45.3) | 39.9 (37.1-42.7) | 36.9 (34.9-38.9) | 37.1 (34.6-39.8) | 39.6 (37.2-42.1) | 41.5 (39.3-43.7) | 39.4 (36.8-42.1) | 42.6 (39.4-45.9) | .01 | 39.5 (38.6-40.3) |
| Arthritis | 22.1(20.3-24.1) | 21.7(19.8-23.7) | 17.5(15.5-19.7) | 25.2(23-27.4) | 26.1(23.3-29.1) | 24.2(22.6-26) | 23.4(20.8-26.2) | 26.8(25.5-28.2) | 27.1(24.9-29.4) | 28.1(25-31.3) | <.01 | 24.5(23.8-25.3) |
| RA | 6.5(5.3-7.8) | 7.4(6.4-8.6) | 6.5(5.4-7.8) | 8.8(7.7-10.1) | 8.7(7.4-10.2) | 8.7(7.7-9.9) | 3.7(3-4.5) | 3.8(3.1-4.7) | 3.8(3-4.8) | 4.2(3.3-5.4) | 0.19 | 6.1(5.8-6.5) |
| OA | 5.6(4.7-6.6) | 4.1(3.4-4.9) | 3.3(2.6-4.2) | 4(3.3-4.7) | 4.2(3.6-5) | 3.9(3.4-4.5) | 11.5(10.1-13.2) | 14.8(13.6-16) | 13.2(12-14.6) | 13.9(12-16) | <.01 | 8.2(7.8-8.7) |
| Diabetes | 8.1 (7.0-9.3) | 8.1 (7.1-9.2) | 9.7 (8.3-11.2) | 9.4 (8.3-10.6) | 10.9 (9.4-12.5) | 10.8 (9.9-11.9) | 11.4 (10.1-12.9) | 12.0 (11.0-13.1) | 13.2 (11.4-15.2) | 13.8 (12.7-15.1) | <.01 | 10.9 (10.5-11.3) |

*N represents unweighted number, and the remaining values are weighted values using NHANES MEC examination weight.

†Figures are expressed as mean ± standard error (for mean age and systolic and diastolic blood pressure), other figures are expressed as percent (95% confidence intervals).

2017 ACC/AHA: American Heart Association/American College of Cardiology (AHA/ACC) 2017 guideline

JNC 7: The Seventh Report of the Joint National Committee on Prevention, Detection, Evaluation, and Treatment of High Blood Pressure

All P for liner trend values were adjusted for age, gender (except gender-specific estimates), race/ethnicity (except race/ethnicity-specific estimates), and body mass index.

**Supplementary Table 2. Competing Risk analysis of the association between hypertension and RA/OA**

|  | **RA** | | **OA** | |
| --- | --- | --- | --- | --- |
|  | **OR (95% CI)** | **P** | **OR (95% CI)** | **P** |
| Hypertension | 2.5(2.25-2.78) | <.01 | 2.68(2.42-2.98) | <.01 |
| Age | 1.29(1.15-1.45) | <.01 | 1.38(1.22-1.55) | <.01 |
| Women | 3.04(2.67-3.45) | <.01 | 3.14(2.77-3.57) | <.01 |
| Mexican American | 2.58(2.11-3.15) | <.01 | 2.88(2.39-3.48) | <.01 |
| Low education level | 2.43(2.09-2.83) | <.01 | 2.91(2.49-3.4) | <.01 |
| Low family income | 2.87(2.31-3.56) | <.01 | 3.08(2.65-3.57) | <.01 |
| Unemployment | 2.2(1.89-2.56) | <.01 | 2.19(1.92-2.51) | <.01 |
| No health insurance | 2.06(1.5-2.83) | <.01 | 2.98(2.21-4) | <.01 |
| BMI | 2.37(2.14-2.64) | <.01 | 2.44(2.21-2.71) | <.01 |
| Inactive physical activity | 2.41(2.09-2.78) | <.01 | 2.85(2.44-3.33) | <.01 |
| Excess Na intake | 2.52(2.11-2.83) | <.01 | 2.67(2.33-2.97) | <.01 |
| Ever cigarette smoking | 1.68(1.41-2) | <.01 | 1.96(1.62-2.39) | <.01 |
| Diabetes | 1.53(1.18-2) | <.01 | 1.66(1.26-2.18) | <.01 |

**Supplementary Table 3. Association between hypertension and arthritis stratified by gender and age**

|  |  | **Crude OR** | **Model 1** | **Model 2** | **Model 3** | **Model 4** |
| --- | --- | --- | --- | --- | --- | --- |
| Age 20-39 | Overall | 1.75 (1.47-2.08) | 1.65 (1.36-1.99) | 1.65 (1.36-1.99) | 1.52 (1.26-1.84) | 1.47 (1.22-1.77) |
| Age 40-59 | Overall | 1.62 (1.47-1.78) | 1.48 (1.34-1.63) | 1.47 (1.34-1.62) | 1.39 (1.26-1.53) | 1.34 (1.22-1.48) |
| Age ≥ 60y | Overall | 1.27 (1.14-1.41) | 1.19 (1.07-1.33) | 1.19 (1.06-1.32) | 1.12 (1.00-1.25)* | 1.10 (0.88-1.18)† |

*P=0.06

†P=0.16

Figures are expressed as odds ratio (95% confidence interval).

Model 1: Adjusted for age, gender, and race

Model 2: Further adjusted for socioeconomic factors including health insurance coverage, education level, employment status and poverty to income ratio

Model 3: Further adjusted for physical activity, excess sodium intake, ever cigarette smoking and obesity

Model 4: Further adjusted for diabetes
